# Hartmann’s procedure in rectal cancer surgery is often an intraoperative decision: a retrospective multicenter study

**DOI:** 10.1007/s00423-024-03237-8

**Published:** 2024-02-07

**Authors:** Elin Mariusdottir, Fredrik Jörgren, Maria Saeed, Jens Wikström, Marie-Louise Lydrup, Pamela Buchwald

**Affiliations:** 1 Department of Surgery, Helsingborg Hospital, Charlotte Yhlens gata 10, 25223 Helsingborg, Sweden; 2 Lund University, Lund, Sweden; 3 Department of Surgery, Skåne University Hospital, Malmö, Sweden; 4 Department of Surgery, Kristianstad Hospital, Kristianstad, Sweden

**Keywords:** Hartmann’s procedure, Intraoperative decision, Rectal cancer

## Abstract

**Purpose:**

This study aimed to investigate patient-related factors predicting the selection of rectal cancer patients to Hartmann’s procedure as well as to investigate how often, and on what grounds, anterior resection is intraoperatively changed to Hartmann’s procedure.

**Methods:**

Prospectively collected data from the Swedish Colorectal Cancer Registry regarding patients with rectal cancer operated upon from January 1 2007 to June 30 2017 in the county of Skåne were retrospectively reviewed. Data were expanded with further details from medical charts. A univariable analysis was performed to investigate variables associated with unplanned HP and significant variables included in a multivariable logistic regression analysis.

**Results:**

Altogether, 1141 patients who underwent Hartmann’s procedure (275 patients, 24%), anterior resection (491 patients, 43%), or abdominoperineal resection (375 patients, 33%) were included. Patients undergoing Hartmann’s procedure were significantly older and had more frequently comorbidity. The decision to perform Hartmann’s procedure was made preoperatively in 209 (76%) patients, most commonly because of a comorbidity (27%) or oncological reasons (25%). Patient preference was noted in 8% of cases. In 64 cases (23%), the decision was made intraoperatively, most often due to anastomotic difficulties (60%) and oncological reasons (22%). Anastomotic difficulties were most often reported due to technical difficulties, a low tumor or neoadjuvant radiotherapy. Male gender was a significant risk factor for undergoing unplanned Hartmann’s procedure.

**Conclusions:**

The decision to perform Hartmann’s procedure was frequently made intraoperatively. Hartmann’s procedure should be considered and discussed preoperatively in old and frail patients, especially in the presence of mid-rectal cancer and/or male gender, since these factors increase the risk of intraoperative anastomotic difficulties.

## Introduction

The standard treatment for rectal cancer in the middle and upper rectum is anterior resection (AR) with total mesorectal excision (TME) [1]. In patients with cancer in the lowest part of the rectum, i.e., ≤ 5 cm from the anal verge, the national Swedish treatment guidelines recommend abdominoperineal resection (APR) to achieve adequate tumor margins [2]. The Hartmann procedure (HP) was originally described by Henri Albert Hartmann in 1921 for the management of rectal cancer [3]. HP is today recommended for patients with a tumor in the middle and upper rectum where the restoration of bowel continuity is associated with an unacceptably high risk of complications due to anastomotic leak (AL) and in patients with a history of incontinence. Other indications for HP could be major adverse intraoperative events [4, 5].

The safety and feasibility of HP was questioned in the early 2000s due to reports on the high risk of pelvic sepsis [6–9]. This caused surgeons to advocate APR to avoid pelvic sepsis related to the rectal remnant, even in tumors above 5 cm from the anal verge. However, recent publications have described lower rates of septic complications suggesting that HP is a reliable alternative [10–12].

The usage of HP varies significantly between countries, reflecting uncertainties regarding indications. The role of HP in rectal cancer treatment is still debated [4, 5]. Furthermore, few studies have reported on the number of patients that are supposed to undergo AR but are changed to HP intraoperatively [13, 14].

The aim of this study was to investigate patient-related factors predicting the selection of rectal cancer patients to HP as well as to investigate how often, and on what grounds, AR is changed to HP intraoperatively.

## Material and methods

### The Swedish ColoRectal Cancer Registry

Since 1996, all patients undergoing resection of rectal cancer in Sweden are registered in the Swedish ColoRectal Cancer Registry (SCRCR). Data on patient and tumor characteristics, diagnostics, treatment, and outcome are registered. The SCRCR has previously been described in detail [15, 16]. The registry has a coverage of > 99% of patients with rectal cancer in Sweden and the internal data validity is high.

The study was approved by the Ethical Review Board of Lund, Sweden (Dnr 2019–01262) and followed the declaration of Helsinki guidelines.

### The study cohort

This was a retrospective study of prospectively collected data from patients undergoing HP, APR, or AR for rectal cancer using prospectively registered data from the SCRCR in the southern part of Sweden from January 1 2007 through June 30 2017.

According to the Swedish national guidelines, the recommended procedure for tumors ≤ 5 cm is APR. All patients with a tumor < 5 cm were excluded from the analysis, but as a considerable portion of patients with a tumor at 5 cm might undergo HP or AR, we included these patients in the study. Furthermore, patients not subjected to resection surgery were excluded. As the group undergoing AR was larger than the HP and APR groups, AR patients were randomly selected on the basis of year of operation 2AR:1HP.

Data from the SCRCR were expanded with further information from medical charts regarding cardiovascular disease, diabetes, pulmonary disease, immunosuppression, and smoking. Preoperative blood tests including albumin, CEA, and creatinine obtained during outpatient visits registered up to 1 month prior to surgery were registered. The preoperative reasons for choosing HP as documented from the outpatient notes and multidisciplinary team conference notes were registered.

If the planned operation according to the outpatient notes was AR and the surgeon decided intraoperatively to perform HP, this was registered as an intraoperative decision. The reasons for the intraoperative changes were collected from the operation notes.

### Definitions

Rectal cancer was defined as adenocarcinoma ≤ 15 cm of the anal verge.

HP was defined as removal of the rectum leaving an anorectal stump.

Emergency rectal resection was defined as a procedure performed during an emergency setting often due to bowel perforation, bleeding, or bowel obstruction.

A colorectal surgeon was defined as a surgeon specialized in colorectal surgery and trained in the TME technique.

### Statistical analysis

Continuous variables are presented as the median with an interquartile range, and categorical data are described using frequencies of counts with associated percentages. Patients were divided into groups based on the operation performed, i.e., HP, AR, or APR. A Shapiro-Wilks test was performed to assess normality. A one-way ANOVA test was used to compare means between the groups in terms of continuous variables and the chi-square test for categorical variables. A Dunnett’s post hoc test was used to identify the means that differ from the reference group (HP).

A separate analysis was performed comparing patients undergoing HP after an intraoperative decision to those in whom the procedure was planned. Nominal variables were compared between groups using Fisher’s exact test and continuous variables were analyzed using a Mann–Whitney test. A univariable analysis was performed to investigate variables associated with unplanned HP and significant variables included in a multivariable logistic regression analysis.

The R version 3.6.1 was used for the analysis, and a *p* < 0.05 was considered significant.

## Results

### Study cohort

**Fig. 1:**
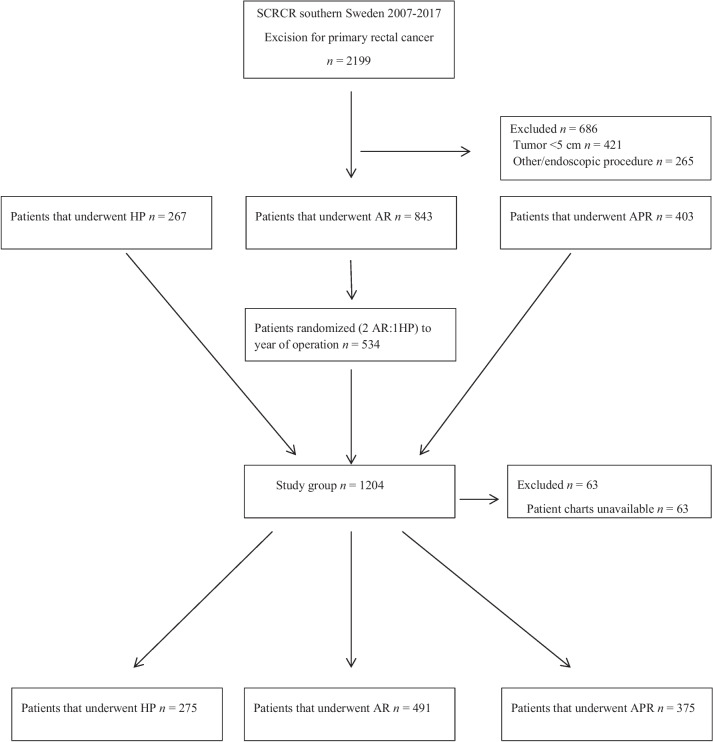
Study flow chart of patients from the SCRCR in the southern part of Sweden from 2007 to 2017. HP, Hartmann’s procedure; AR, anterior resection; APR, abdominoperineal resection

During the period, a total of 2199 patients with rectal cancer were registered in the SCRCR in the southern part of Sweden. Altogether, 421 patients with a tumor height < 5 cm were excluded as were 265 patients undergoing endoscopic or local excision. After random selection, 534 AR patients were included and the remaining 309 were excluded. This resulted in 1204 patients who were accessible for inclusion (Fig. 1).

A review of the medical records revealed that 19 patients registered as AR and two patients registered as APR had undergone HP. Another 63 patients were excluded due to missing medical records.

During the study period, HP was performed in 275 patients (19%) with AR performed in 800 patients (55%) and 375 (26%) undergoing APR. As 309 AR patients were randomly excluded the study cohort consisted of 1141 patients: 275 (24%) HP, 491 (43%) AR, and 375 (33%) APR (Fig. 1).

### Demographics of the study cohort

Demographics of the study cohort are presented in Table 1. When comparing the HP group to AR and APR patients, patients undergoing HP were significantly older (*p* < 0.001). There was a significant gender difference with 59% males in the HP and AR groups compared to 68% in the APR group (*p* = 0.02). HP patients more frequently reported a history of cardiovascular disease and diabetes, and had a higher ASA score compared to the AR and APR groups (*p* < 0.001). The preoperative albumin was lower in the HP group compared to the AR group (*p* < 0.001) but not compared to the APR group (*p* = 0.14). The median tumor height was lower in the APR group, 7 cm compared with 10 cm in the HP group and AR group (*p* < 0.001) and 222 patients with a tumor height 6–10 cm underwent APR. Neoadjuvant radiotherapy (RT) was used less frequently in the HP group.

**Table 1:** Patient, tumor, and treatment characteristics of patients with rectal cancer treated with Hartmann’s procedure, anterior resection, or abdominoperineal resection in the study cohort

	All patients (*n* = 1141)	HP (*n* = 275)	AR (*n* = 491)	APR (*n* = 375)	*p* value
Age (years) at surgery*	70 (14)	75 (14)	67 (13)	70 (14)	< 0.001
Male gender	708 (62)	162 (59)	292 (59)	254 (68)	0.02
BMI > 30 (kg/m^2^)	172 (15)	44 (16)	72 (15)	56 (15)	0.75
Cardiovascular disease	558 (49)	160 (58)	202 (41)	196 (52)	< 0.001
Diabetes mellitus	149 (13	45 (16)	50 (10)	54 (14)	0.03
Pulmonary disease	100 (9)	30 (11)	33 (7)	37 (10)	0.09
Immunosuppression	48 (4)	16 (6)	16 (3)	16 (4)	0.23
Smoking history					0.20
Never/former	994 (89)	228 (87)	442 (91)	324 (88)	
Current	121 (11)	35 (13)	44 (9)	43 (12)	
ASA score					< 0.001
I–II	842 (74)	159 (58)	424 (87)	259 (70)	
III–IV	285 (26)	112 (42)	61 (13)	112 (30)	
Albumin (g/L)*	37 (5)	36 (4.5)	38 (4)	36 (5)	< 0.001
CEA (µg/L) *	3 (6)	4 (10)	3 (4)	4 (5.5)	0.66
Creatinine (µmol/L) *	77 (24)	78 (29)	76 (21)	76 (26)	0.12
Tumor height (cm)					< 0.001
Low 5	128 (11)	6 (2)	8 (2)	114 (31)	
Mid 6–10	625 (55)	143 (52)	260 (53)	222 (59)	
High 11–15	388 (34)	126 (46)	223 (45)	39 (10)	
Preoperative T stage					
cT1-2	254 (22)	64 (23)	126 (26)	64 (17)	0.007
cT3	632 (56)	138 (51)	281 (57)	213 (57)	0.19
cT4	156 (14)	43 (16)	43 (9)	70 (19)	< 0.001
cTx	82 (8)	23 (9)	33 (8)	26 (7)	0.64
TNM stage					0.26
I–II	596 (54)	136 (50)	266 (56)	194 (54)	
III–IV	512 46)	137 (50)	209 (44)	166 (46)	
Neoadjuvant RT	730 (64)	137 (50)	303 (62)	290 (78)	< 0.001
Neoadjuvant CHT	254 (22)	61 (22)	90 (18)	103 (28)	0.006
Emergency surgery	16 (1.4)	12 (4)	3 (0.6)	1 (0.2)	< 0.001
Preoperative bowel preparation	667 (59)	123 (45)	433 (88)	111 (30)	< 0.001
Surgical competence					0.83
Colorectal	937 (82)	225 (82)	407 (83)	305 (81)	
General	155 (14)	41 (15)	53 (11)	61 (16)	
Missing values	49 (4)	9 (3)	31 (6)	9 (3)	
Minimal invasive surgery	201 (18)	33 (12)	102 (21)	66 (18)	0.04
Conversion to open surgery	40 (20)	12 (36)	19 (19)	9 (14)	0.34
Intraoperative perforation	64 (6)	14 (5)	8 (2)	42 (11)	< 0.001
Rectal washout	725 (64)	215 (79)	469 (96)	41 (11)	< 0.001
Operation time (min)*	312 (145)	251 (152)	297 (97)	378 (167)	< 0.001
Blood loss*	400 (500)	400 (489)	300 (350)	500 (500)	< 0.001

Values in parenthesis are % unless * representing median with interquartile range

*HP* Hartmann’s procedure, *AR* anterior resection, *APR* abdominal perineal resection, *BMI* body mass index, *CEA* carcinoembryonic antigen, *ASA* American Society of Anesthesiologists, *TNM* tumor node metastasis staging system, *RT* radiotherapy, *CHT* chemotherapy

Emergency surgery was performed in 16 (1.4%) patients in the study; 12 patients underwent HP. Of the patients undergoing AR, 428 (87%) received a diverting stoma.

### Preoperative reasons for performing HP

The decision to perform HP was made preoperatively in 209 patients (76%). Reasons are presented in Table 2. The most common cause for performing HP was comorbidity in 57 cases (27%). In 53 cases (25%), the reason was oncological, most often because of planned adjuvant treatment or a locally advanced tumor requiring multi-visceral surgery. In 24 cases (11%), high age was the rationale for performing HP, while patient preference was stated in 17 cases (8%). Incontinence was the sole reason for choosing HP in five patients (2%).

**Table 2:** The preoperative reasons to perform Hartmann´s procedure as stated in the multi-disciplinary team conference note and the preoperative outpatient visit

Preoperative reasons to perform HP	(*n* = 209)
Comorbidity	57 (27)
Oncology-related Advanced tumor — planned adjuvant treatment Advanced tumor — locally advanced Advanced tumor — palliation	53 (25) 28 22 3
High age	24 (11)
Patient preference	17 (8)
High age and comorbidity	16 (8)
Emergency surgery	7 (3)
Low tumor	6 (3)
Comorbidity and incontinence	6 (3)
Incontinence alone	5 (2)
High age and incontinence	5 (2)
Reason not clearly stated	13 (6)

Values in parenthesis are %

*HP* Hartmann’s procedure

### Intraoperative decision to perform HP

Altogether, 64 patients (23%) were planned for AR but the decision was changed to HP during the operation. In 38 patients (60%), anastomotic difficulties resulted in an unsafe anastomosis. The surgeon reported technical difficulties such as stapling device malfunction or too short an anastomotic distance from the anal verge. In eight cases, the tissue damage caused by RT resulted in an unsafe anastomosis, while in 14 patients (22%), the reason was oncological factors: e.g., a more advanced tumor than expected caused an intraoperative change of plan. In five cases (8%), intraoperative bowel perforation occurred, resulting in HP. The reasons for intraoperative change are presented in Table 3. Two patients (1%) were planned to undergo APR but were intraoperatively changed to HP because of a tumor further from the anal verge than previously assessed.

**Table 3:** Reasons for intraoperative decision to change from anterior resection to Hartmann’s procedure according to the surgeon

Intraoperative reasons to perform HP	(*n* = 64)
Anastomosis related Technical difficulties Anastomosis too low RT tissue damage Diminished blood flow to the bowel	38 (59) 14 12 8 4
Oncology related Advanced tumor — planned adjuvant treatment Advanced tumor — palliation Major bleeding Bowel perforation Reason not clearly stated	14 (22) 12 2 5 (8) 5 (8) 2 (3)

Values in parenthesis are %

*HP* Hartmann’s procedure, *RT* radiotherapy

A comparison of patients who were supposed to undergo AR but had this changed to HP intraoperatively and those undergoing preoperatively planned HP is presented in Table 4. Patients in the unplanned HP group were younger than those undergoing planned HP (71 years compared with 77 years; *p* = 0.002). Moreover, male gender (73% vs 55%;* p* = 0.01), lower ASA (*p* = 0.02), and higher rate of neoadjuvant RT (63% vs 46% *p* = 0.02) were more often observed in the unplanned vs the planned HP group.

**Table 4:** Comparison between patients undergoing planned Hartmann’s procedure with patients where the decision to perform Hartmann’s procedure was made intraoperatively

	Planned HP (*n* = 209)	Unplanned HP (*n* = 64)	*p* value
Age (years) at surgery*	77 (14)	71 (13)	0.002
Male gender	114 (55)	47 (73)	0.01
BMI > 30 (kg/m^2^)	29 (14)	15 (23)	0.08
Comorbidity			
Cardiovascular disease	120 (57)	40 (63)	0.56
Diabetes mellitus	32 (15)	13 (20)	0.45
ASA score			
I–II	111 (53)	46 (73)	0.02
III–IV	95 (47)	17 (27)	
Albumin (g/L)*	36 (4)	37 (5)	0.31
CEA (µg/L) *	4 (9)	5 (9)	0.03
Creatinine (µmol/L) *	78 (30)	80 (24)	0.15
Tumor height (cm)			0.23
Low 5	6 (3)	0 (0)	
Mid 6–10	103 (49)	38 (59)	
High 11–15	101 (48)	26 (41)	
Pre-operative T stage			
cT1-2	54 (26)	10 (16)	0.09
cT3	99 (48)	39 (61)	0.06
cT4	33 (16)	10 (16)	0.98
cTx	19 (10)	4 (7)	0.67
TNM stage			1
I–II	104 (50)	31 (48)	
III–IV	104 (50)	32 (50)	
Missing values	0	1 (2)	
Neoadjuvant RT	96 (46)	40 (63)	0.02
Neoadjuvant CHT	47 (22)	17 (27)	0.30

Values in parenthesis are % unless * representing median with interquartile range

*HP* Hartmann’s procedure*. BMI* body mass index*, CEA* carcinoembryonic antigen. *ASA* American Society of Anesthesiologists. *TNM* Tumor Node Metastasis staging system. *RT* radiotherapy. *CHT* chemotherapy

Results from the multivariable analysis showed that male gender increased the likelihood of intraoperative changes: OR 2.45 (CI:1.31–4.79) as well as ASA score I and II: OR 2.07 (CI:1.10–4.01). Neoadjuvant RT was not a significant risk factor after correcting for age, gender, and ASA score (Table 5).

**Table 5:** Univariable and multivariable logistic regression analyzing risk factors for intraoperative change from anterior resection to Hartmann’s procedure

	Univariable analysis			Multivariable analysis		
Variables	OR	95% CI	*p* value	OR	95% CI	*p* value
Age (years)	0.95	0.92–0.99	0.008	0.96	0.93–0.98	0.006
Male gender	2.30	1.26–4.37	0.008	2.45	1.31–4.79	0.006
BMI > 30 (kg/m^2^)	1.87	0.91–3.73	0.08			
ASA I-II	2.16	1.19–4.07	0.01	2.07	1.10–4.01	0.03
CEA (µg/L)	1.00	1.00–1.01	0.007	1.00	0.99–1.00	0.15
Neoadjuvant RT	1.96	1.11–3.52	0.02	1.60	0.80–3.27	0.19

*BMI* body mass index, *CEA* carcinoembryonic antigen, *ASA* American Society of Anesthesiologists, *RT* radiotherapy

## Discussion

The current article investigates the indications for performing HP in a large cohort of rectal cancer patients in whom it theoretically had been technically possible to perform AR. Reasons behind the preoperative decision to perform HP are in line with Swedish guidelines but an intraoperative decision to change from AR to HP was made in 23% of cases, most often due to anastomotic difficulties.

A comparison of patients undergoing HP with patients undergoing AR and APR reveals that the reasons for performing HP are multifactorial, including higher age and ASA score, lower preoperative albumin, and more frequently a history of cardiovascular disease and diabetes.

In the present study, comorbidity is the leading cause for choosing HP preoperatively (27%). Oncological reasons or the high risk of AL are stated in 25% of patients, such as planned adjuvant treatment or locally advanced tumors. High age is stated as the reason in 11%, which is consistent with previous studies. A recent meta-analysis comparing HP to APR including intersphincteric APR (iAPR) found that the main indications for nonrestorative bowel surgery were comorbidities, advanced age, and tumor stage. HP was more often used in older and frailer patients who were less fit for surgery, complicating comparisons [9]. Studies comparing HP with iAPR also conclude that comorbidities are the most common reason for nonrestorative bowel surgery as well as the high risk of AL [14, 17].

Poor sphincter function is often mentioned as an indication for performing HP [4, 18, 19]. Interestingly, our study revealed that incontinence alone is stated as the main reason for HP in only 2% of cases, with another 5% undergoing HP because of a combination of comorbidities and incontinence. The reasons for this are not clear, a possible explanation may be incomplete medical charts. However, since patient preference was the reason for performing HP in 8% of cases, this suggests that surgeons discuss treatment options with patients.

It is important for the patient and surgeon to decide upon a surgical strategy preoperatively to prevent intraoperative alterations. Very few studies discuss the intraoperative decision to change from AR to HP. We find that the most common reasons for choosing HP intraoperatively are difficulties related to the anastomosis. Moreover, numerically conversions were more frequently carried out in the unplanned HP group. Not surprisingly, 73% of the patients undergoing unplanned HP are male, who have a narrower pelvis, making stapling more challenging [20]. Furthermore, the multivariable analysis showed that males had a higher risk of undergoing unplanned HP compared to females.

Altogether 222 patients with a tumor 6–10 cm from the anal verge underwent APR, and this likely reflects the fact that many surgeons refrain from using HP in cases where AR is considered unsafe as previous studies have reported higher risk of complications following HP [6, 7].

The fact that patients undergoing unplanned HP were younger and had a lower ASA score probably reflects that surgeons are much more likely to choose AR in this patient group and unforeseen events cause the change of plan.

The risk of AL is increased in tumors close to the anal verge and the functional results of a low anastomosis are poorer [20, 21]. Surgeons performed HP instead of AR in 12 cases because the anastomosis was deemed too low. The univariable analysis showed no significant difference in tumor height in the unplanned vs planned group. This may be due to small sample size or that patients with tumor 6–10 cm from the anal verge were grouped together. Another important aspect is that decision-making is entirely at the discretion of the responsible surgeon and what constitutes a too low anastomosis is difficult to define.

Another major reason for not performing AR as planned is an intra-operative finding of a more advanced tumor than previously assessed. No significant difference was seen in the pre-operative T stage in the unplanned vs planned HP group; this may reflect difficulties in staging. A national study from a Swedish cohort shows that the accuracy of MRI in preoperative staging is lower than expected [22]. Advanced tumor stage has been shown to increase the risk of AL, and avoiding the risk of anastomotic complications ensures adjuvant treatment without delay [20, 21].

## Limitations

One limitation of this study is its retrospective design, with difficulties assessing reasons for choosing HP in some cases, since notes are retrospectively reviewed. Another limitation is that patients undergoing AR were randomized as this group was much larger. However, since the ratio HP:AR was 1:2, this probably did not affect the result. In the present study, the rate of patients undergoing minimally invasive surgery was low (18%), probably explained by that the study period was from 2007 to 2017. For the same reason, other novel operative techniques such as iAPR, transanal total mesorectal excision, or transanal transection with single-stapled anastomosis could not be assessed in the study [23]. The rate of minimally invasive surgery has steadily increased in Sweden from 2015 and new surgical methods introduced [24].

Overall, the large study cohort with a complete data set resulted in reliable results.

Our findings provide valuable insight into the treatment of elderly and frail patients. A lower risk of reoperation and septic complications in HP compared to AR has been reported; the authors conclude that HP should be considered in older frail patients [5]. This is important as the number of elderly patients with rectal cancer has increased in recent years, which needs to be taken into consideration when constructing guidelines regarding rectal cancer treatment [25].

A large study on the risk of complications after rectal cancer surgery as well as assessing quality of life after surgery comparing HP with AR and APR would increase our knowledge on the topic. Furthermore, it is important to compare oncological outcomes after HP, AR, and APR.

## Conclusions

HP is most often performed in frail patients where the risk of AL is significant. Another common reason is locally advanced tumors with planned adjuvant treatment. Incontinence alone is seldom the reason for choosing HP.

A significant number of patients undergo HP as a result of an intraoperative decision. To the best of our knowledge, this is the first detailed study on intraoperative changes from AR to HP giving valuable insight into risk factors. Our findings suggest that male gender, a distal transection line close to the anal sphincter, and a more advanced tumor may increase the risk of anastomotic difficulties resulting in an unplanned HP. Surgeons should consider HP preoperatively in these cases.

## Data Availability

The datasets used during the current study are available from the corresponding author on reasonable request.

## Abbreviations

*TME*: Total mesorectal excision
*AR*: Anterior resection
*APR*: Abdominoperineal resection
*iAPR*: Intersphincteric APR
*HP*: Hartmann’s procedure
*AL*: Anastomotic leak
*SCRCR*: Swedish ColoRectal Cancer Registry
*CEA*: Carcinoembryonic antigen
*ASA score*: American Society of Anesthesiologists
*RT*: Radiotherapy
*CHT*: Chemotherapy
*BMI*: Body mass index
*TNM*: Tumor node metastasis staging system
